# Associations of acrylamide with non-alcoholic fatty liver disease in American adults: a nationwide cross-sectional study

**DOI:** 10.1186/s12940-021-00783-2

**Published:** 2021-08-30

**Authors:** Zhening Liu, Jinghua Wang, Shenghui Chen, Chengfu Xu, Yu Zhang

**Affiliations:** 1 Department of Gastroenterology, The First Affiliated Hospital, Zhejiang University School of Medicine, 79 Qingchun Road, Hangzhou, 310003 Zhejiang China; 2 Zhejiang Key Laboratory for Agro-Food Processing, Fuli Institute of Food Science, Department of Food Science and Nutrition, College of Biosystems Engineering and Food Science, Zhejiang University, 866 Yuhangtang Road, Hangzhou, 310058 Zhejiang China

**Keywords:** Fatty liver disease, Acrylamide, Association, Risk

## Abstract

**Background:**

Acrylamide (AA) is a toxicant to humans, but the association between AA exposure and the risk of non-alcoholic fatty liver disease (NAFLD) remains unclear. In this study, our objective is to examine the cross-sectional association between AA exposure and the risk of NAFLD in American adults.

**Methods:**

A total of 3234 individuals who took part in the National Health and Nutrition Examination Survey (NHANES) 2003–2006 and 2013–2016 were enrolled in the study. NAFLD was diagnosed by the U.S. Fatty Liver Index. Multivariable logistic regression models were applied to estimate the association between AA and NAFLD in the whole group and the non-smoking group.

**Results:**

We discovered that in the whole group, serum hemoglobin adducts of AA (HbAA) were negatively associated with the prevalence of NAFLD after adjustment for various covariables (*P* for trend < 0.001). Compared with individuals in the lowest HbAA quartiles, the odds ratios (ORs) with 95% confidence intervals (CIs) in the highest HbAA quartiles were 0.61 (0.46–0.81) and 0.57 (0.36–0.88) in the whole group and the non-smoking group, respectively. In contrast, HbGA/HbAA showed a significantly positive correlation with the prevalence of NAFLD in both groups (*P* for trend < 0.001). In addition, HbGA was not significantly associated with NAFLD in the whole group or the non-smoking group.

**Conclusions:**

HbAA is negatively associated with NAFLD whereas HbGA/HbAA is positively associated with NAFLD in adults in the U.S. Further studies are needed to clarify these relationships.

## Background

Non-alcoholic fatty liver disease (NAFLD) is one of the most common chronic liver disorders, with its global prevalence estimated to be 24%, a figure expected to rise rapidly in the future [1]. NAFLD is characterized as a spectrum ranging from simple steatosis, to steatohepatitis, fibrosis, and even hepatocellular carcinoma, and it is strongly associated with metabolic syndrome [2]. Current data confirm that NAFLD is a complex disease involving multiple environmental factors and genetic predispositions [3].

Acrylamide (AA, C_3_H_5_NO), a highly reactive α, β-unsaturated carbonyl compound, is widely used in the production of its polymer, polyacrylamide, for industrial processes and gel electrophoresis in laboratories [4]. AA is generated through the Maillard reaction between asparagine and reducing sugars in low moisture-processed, carbohydrate-rich food cooked at high temperatures (above 120 °C), such as French fries and biscuits [5]. Besides food, cigarette smoking is another crucial cause of exposure, with the level of AA ranging from 0.5 to 4.2 μg per cigarette [6]. After being absorbed into the human body, AA can be metabolized by either reacting with cytochrome P-450 2E1 (CYP2E1) to form a reactive epoxy compound glycidamide (GA) or conjugating with glutathione (GSH) to form GSH conjugates [7]. Like glucose, AA and GA are also able to react with hemoglobin to form hemoglobin adducts of AA (HbAA) and GA (HbGA). Considering the lifespan of erythrocytes, these two adducts can be adopted as proper biomarkers to evaluate AA exposure within the past 4 months [8].

In 1994, AA was defined as a Group 2A carcinogenic compound by the International Agency for Research on Cancer (IARC) [9], although its carcinogenicity remains inconsistent and ambiguous across different epidemiological studies. In 2002, AA was discovered in food by Swedish scientists, which sparked research on its hazard level [10]. Similar to classic endocrine-disrupting chemicals (EDC), AA also has the potential to interfere with the endocrine system by impairing endogenous hormonal activity [11]. Previous studies showed that high AA exposure is associated with obesity-related outcomes in a population of U.S. adults [12]. Obesity has been linked with NAFLD independently of other metabolic factors [13]. It plays a key role in not only the onset of liver steatosis but also its progression [14]. Moreover, the severity of NAFLD and mortality in NAFLD patients also increase with obesity [15–17]. Besides obesity, insulin resistance has also been shown to be closely related to NAFLD [18]. Hepatic insulin action is required for lipid synthesis, glucose production and the development of hepatic steatosis during insulin resistance [19]. HbAA was shown to be associated with reduced serum insulin levels [20] and the risk of diabetes [21] in previous NHANES research. Furthermore, an in vivo study also indicated that AA disrupted glucose homeostasis in female rats possibly by impairing the physiological effects of insulin [22]. However, no epidemiological evidence has been reported to support the association between AA exposure and NAFLD.

In this study, we analyzed the associations of HbAA and HbGA with NAFLD among a nationally representative sample of American adults who were enrolled in the National Health and Nutrition Examination Survey (NHANES) 2003–2006 and 2013–2016.

## Methods

### Study design and population

**Fig. 1:**
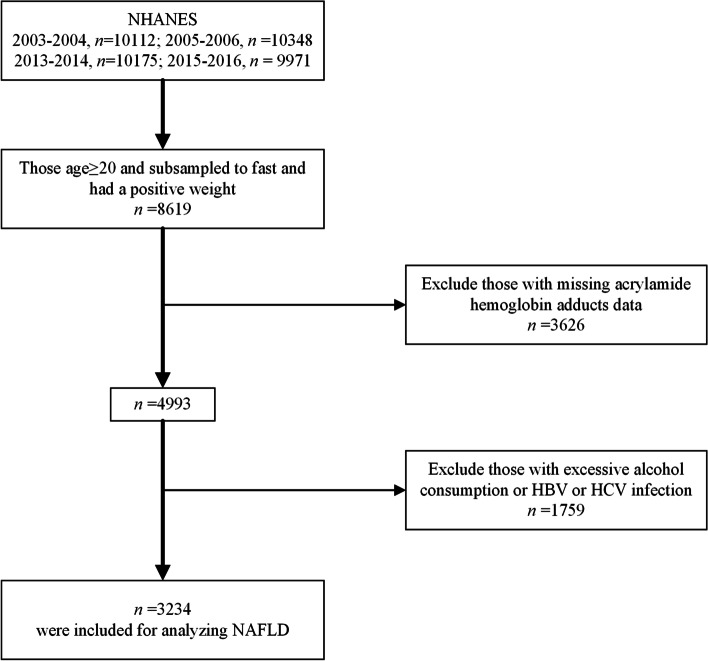
Flow diagram of inclusion criteria from NHANES 2003–2006 and 2013–2016. HBV: hepatitis B virus; HCV: hepatitis C virus

The NHANES is a series of cross-sectional surveys conducted every 2 years to continuously measure the health and nutritional status of adults and children, covering different population groups and health topics in the United States. In our study, we used the publicly available data of NHANES 2003–2006 and 2013–2016, in which HbAA and HbGA levels were tested. A total of 40,606 individuals who participated in the four survey cycles were considered in this study. Of these participants, 8619 participants aged over 20 years were subsampled to fast before attending a morning exam session, among which 4993 participants were eligible for analysis of HbAA and HbGA. We also excluded those with alcohol consumption > 2 drinks/day for men or > 1 drink/day for women, and those infected with hepatitis B virus (HBV) or hepatitis C virus (HCV) (*n* = 1759). Finally, 3234 participants were enrolled as the study population (Fig. 1).

### Analysis of acrylamide biomarkers

The laboratory procedure for acrylamide biomarkers has been described in detail in the NHANES documentation [23]. Briefly, after the measurement of total hemoglobin, the Edman products of N-terminal HbAA and HbGA in human whole blood or erythrocytes were isolated and determined through high-performance liquid chromatography tandem mass spectrometry (HPLC/MS/MS). Final results are reported as pmol adduct per g hemoglobin (pmol/g Hb).

### Definitions of NAFLD and other metabolic disorders

The ascertainment of NAFLD is based on the U.S. fatty liver index (US FLI), which was first introduced by Ruhl et al. [24]. The US FLI was established on the NHANES 1988–1994 data and calculated by using the following factors: ethnicity (non-Hispanic black, Mexican American), age (years), gamma glutamyl transferase (U/L), waist circumference (cm), fasting insulin (pmol/L), and fasting glucose (pmol/L). A US FLI ≥ 30 was selected to rule in fatty liver as suggested, and those with excessive alcohol consumption (> 2 drinks/day for men or > 1 drink/day for women) were excluded [24].

In addition, body mass index (BMI) was calculated as weight divided by height squared (kg/m^2^) and used to define obesity (BMI ≥ 30.0 kg/m^2^) [25]. Hypertension was defined as systolic blood pressure exceeding 140 mmHg or diastolic blood pressure exceeding 90 mmHg or the use of anti-hypertensive agents [26]. Type 2 diabetes mellitus (T2DM) was defined as high fasting plasma glucose (≥ 126 mg/dL) or high glycated hemoglobin (HbA1c, ≥ 6.5%) or the use of anti-hyperglycemic agents [27]. Hyperuricemia was defined as serum uric acid levels ≥ 420 μmol/L in men and ≥ 360 μmol/L in women [28].

### Covariables

We considered age (years), gender (man/woman), race/ethnicity (non-Hispanic white, non-Hispanic black, Mexican American, and other), education level (under high school, high school, and above high school), family income-to-poverty ratio (PIR), marital status (married/other), serum cotinine level, total fat intake (gm), total energy intake (kcal), albumin (g/L), LDL-cholesterol (mmol/L), total cholesterol (mmol/L), and platelet count (1000 cells/μL) as covariables. Values of total fat intake and total energy intake were obtained from 24-h dietary recall interviews.

### Statistical analysis

Due to the complex, multistage sampling design used to select participants in NHANES, we constructed 8-year fasting subsample MEC weights (WTSAF2YR*1/4) for combined survey cycles (2003–2006 and 2013–2016). Surveymeans and Surveyfreq procedures were performed to describe variables in weighted forms. Continuous variables were compared by using the Surveyreg procedure, and categorical variables were compared by using the Rao–Scott *χ*^2^ test as recommended [29]. A quartile-based analysis was carried out by dividing HbAA and HbGA levels into quartiles, and the lowest one was set as the reference. Then, the Surveylogistic regression model was used to evaluate the risk factors of NAFLD. We also applied increasing degrees of adjustment to the three models. Model 1 was only adjusted for demographic and social-economic factors (age, gender, race/ethnicity, education level, family PIR, marital status, and serum cotinine level). Model 2 was adjusted for factors in model 1 plus dietary factors (total fat intake and total energy intake). Model 3 was further adjusted for biochemistry factors. The NOMCAR option was used in the estimation procedures to treat missing values as not missing completely at random. Furthermore, we conducted mediation analysis to assess whether the association between AA and NAFLD was mediated by BMI, insulin, fasting glucose or blood pressure by applying the publicly available SAS macro *%mediate* [30]. SAS 9.4 (SAS Institute Inc., Cary, NC) was employed for all analyses in this study. Two-sided *P* < 0.05 was considered to be statistically significant.

## Results

From NHANES 2003–2006 and 2013–2016, a total of 3234 participants, 49.6% men and 50.4% women, were analyzed in our study. All the participants were divided into quartiles based on their HbAA levels as shown in Table 1. There were 70.0% non-Hispanic whites, 11.3% non-Hispanic blacks, 7.8% Mexican Americans, and 11.0% participants from other races. Compared with quartiles 1–3, the proportion of smokers soared to 62.2% in quartile 4, while the marriage rate decreased to 53.6%. Besides, those with higher levels of HbAA tended to be younger and have a lower BMI but higher energy intake and total fat intake. Interestingly, fasting triglyceride levels decreased monotonically in quartiles 1–3, but rose again in quartile 4, demonstrating a U-shaped curve, whereas HDL-C showed the opposite tendency. Furthermore, 30.8% of the participants had NAFLD according to their US FLI scores, and NAFLD prevalence was negatively associated with HbAA levels (*P* = 0.001). In subgroups, the prevalence of NAFLD grew by 2.5-fold and 4.5-fold, respectively, in the elevated alanine aminotransferase (ALT) group (ALT > 40 U/L) and the obese group (BMI ≥ 30 kg/m^2^). These results indicated a significant association of HbAA level with NAFLD and metabolic abnormalities.

**Table 1:** Clinical characteristics of the participants in NHANES 2003–2006 according to HbAA quartiles

Variables	Total (*n* = 3234)	HbAA quartiles				
		Q1	Q2	Q3	Q4	*P*
Age, year	49.75 ± 0.55	52.26 ± 0.88	50.94 ± 0.80	49.48 ± 0.75	46.64 ± 0.63	< 0.001
Male, %	49.6 (1.0)	45.4 (2.0)	44.1 (2.2)	47.8 (2.2)	60.2 (1.6)	< 0.001
Race/ethnicity, %						< 0.001
Non-Hispanic White	70.0 (2.1)	73.0 (2.6)	68.9 (2.1)	72.0 (2.8)	70.0 (2.1)	
Non-Hispanic Black	11.3 (1.2)	10.1 (1.2)	11.1 (1.5)	13.6 (1.6)	11.3 (1.2)	
Mexican American	7.8 (1.0)	8.9 (1.4)	9.6 (1.1)	6.4 (1.3)	7.8 (1.0)	
Others	11.0 (0.8)	8.0 (1.2)	10.4 (1.4)	8.0 (1.1)	11.0 (0.8)	
Smokers, %	20.9 (1.2)	5.4 (1.1)	6.2 (1.4)	7.0 (1.0)	62.2 (2.2)	< 0.001
Marriage, %	63.9 (1.1)	67.8 (1.7)	67.3 (2.8)	67.8 (2.1)	53.6 (1.8)	< 0.001
Education levels, %						< 0.001
Under high school	17.1 (1.1)	17.6 (1.5)	14.5 (1.5)	15.1 (1.7)	21.0 (2.0)	
High school	25.0 (1.1)	23.4 (2.2)	22.4 (2.2)	23.2 (1.8)	30.5 (1.9)	
Above high school	58.0 (1.5)	59.0 (1.8)	63.1 (2.4)	61.6 (2.3)	48.6 (2.7)	
Family PIR	3.07 ± 0.04	2.96 ± 0.06	3.15 ± 0.06	3.31 ± 0.06	2.86 ± 0.06	< 0.001
Body mass index, kg/m^2^	29.09 ± 0.16	29.61 ± 0.22	29.64 ± 0.30	28.72 ± 0.35	28.46 ± 0.30	0.036
Systolic blood pressure, mmHg	123.23 ± 0.47	125.15 ± 0.81	123.6 ± 0.85	122.34 ± 1.11	122.07 ± 0.74	0.059
Diastolic blood pressure, mmHg	70.08 ± 0.32	70.41 ± 0.90	69.88 ± 0.53	70.27 ± 0.52	69.81 ± 0.60	0.846
Albumin, g/L	42.25 ± 0.10	42.23 ± 0.17	42.14 ± 0.15	42.34 ± 0.13	42.27 ± 0.15	0.708
Alanine aminotransferase, U/L	25.26 ± 0.54	24.97 ± 1.42	24.92 ± 0.68	26.37 ± 0.54	24.78 ± 0.48	0.151
γ-Glutamyl transpeptidase, U/L	25.64 ± 0.54	26.58 ± 1.11	24.73 ± 1.11	25.02 ± 1.06	26.29 ± 1.16	0.420
Fasting triglyceride, mg/dl	139.79 ± 2.60	144.28 ± 5.49	144.94 ± 5.71	124.59 ± 4.06	145.45 ± 6.16	0.008
Total cholesterol, mmol/L	5.15 ± 0.03	5.14 ± 0.05	5.19 ± 0.06	5.14 ± 0.04	5.14 ± 0.05	0.883
HDL-C, mmol/L	1.40 ± 0.01	1.39 ± 0.02	1.42 ± 0.02	1.47 ± 0.02	1.31 ± 0.02	< 0.001
LDL-C, mmol/L	3.00 ± 0.02	2.94 ± 0.03	3.00 ± 0.04	2.99 ± 0.04	3.06 ± 0.04	0.165
Platelet count, 1000 cells/μL	259.26 ± 1.86	252.75 ± 2.67	258.53 ± 4.10	259.82 ± 2.46	265.19 ± 3.12	0.047
Fasting blood glucose, mmol/L	5.82 ± 0.04	5.98 ± 0.05	5.88 ± 0.08	5.68 ± 0.05	5.77 ± 0.08	0.002
Total energy intake, kcal	2150.57 ± 20.29	2001.84 ± 29.72	2111.09 ± 48.95	2165.01 ± 27.59	2305.21 ± 41.17	< 0.001
Total fat intake, gm	84.14 ± 0.84	78.44 ± 1.42	82.01 ± 2.06	85.92 ± 1.85	89.45 ± 1.9	< 0.001
HbGA/HbAA	0.95 ± 0.02	1.04 ± 0.05	0.98 ± 0.02	0.95 ± 0.02	0.82 ± 0.02	< 0.001
USFLI	24.04 ± 0.46	26.61 ± 0.76	25.89 ± 1.03	21.52 ± 1.03	22.4 ± 0.83	0.002
NAFLD, %	30.8 (1.0)	34.7 (1.8)	34.0 (2.2)	25.4 (1.8)	29.5 (1.8)	0.001
**Subgroup, NAFLD prevalence**						
**BMI**						
< 30 kg/m^2^	13.1 (0.8)	14.5 (1.9)	15.5 (1.9)	9.8 (1.1)	13.0 (1.4)	0.056
≥ 30 kg/m^2^	61.8 (1.7)	65.4 (2.5)	61.5 (3.7)	57.4 (3.1)	62.4 (3.7)	0.408
**ALT**						
≤ 40 U/L	26.9 (1.0)	29.9 (2.0)	29.7 (2.2)	20.7 (1.7)	27.2 (1.8)	0.002
> 40 U/L	69.1 (2.6)	81.4 (5.1)	75.0 (6.0)	63.6 (5.6)	56.4 (6.9)	0.016

Participants were classified into quartiles according to HbAA levels: Q1, HbAA ≤ 36.10 pmol/g Hb; Q2, 36.10 < HbAA ≤ 47.60 pmol/g Hb; Q3, 47.60 < HbAA ≤ 66.50 pmol/g Hb; and Q4, HbAA > 66.50 pmol/g Hb

Continuous variables were presented as the weighted mean ± SE and compared by Survey regression models

Categorical variables were presented as weighted percent (SE) and compared by using the Rao–Scott χ^2^ test

**Fig. 2:**
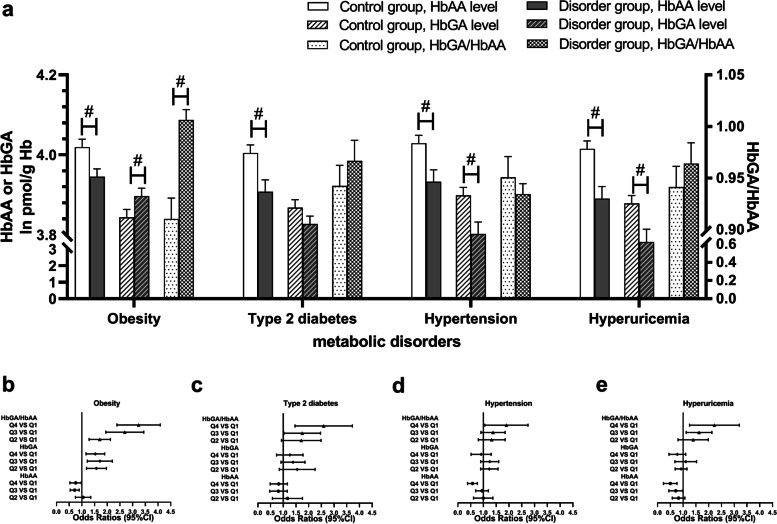
HbAA and HbGA levels in participants with metabolic disorders. **a** HbAA, HbGA levels (log_e_ transformed) and their ratio (HbGA/HbAA) in both case groups and control groups. **b**-**e** Association of AA biomarkers with the risk of metabolic disorders after adjusting for age, gender, race/ethnicity, education levels, family PIR, marital status, serum cotinine level, total fat intake, total energy intake, serum albumin, LDL-cholesterol, total cholesterol and blood platelet count. **#**: *P* < 0.01, compared by Survey regression models

Obesity and related metabolic disorders, including type 2 diabetes, hypertension, and hyperuricemia, are all significantly associated with NAFLD as previously reported [31, 32]. Therefore, we explored the association between AA biomarkers and these metabolic disorders. As shown in Fig. 2a, HbAA concentrations were significantly lower in all disorder groups than in healthy controls. On the contrary, HbGA levels were significantly higher in the obese group but lower in hypertension and hyperuricemia groups. Correspondingly, a significantly higher ratio of HbGA and HbAA was discovered in the obese group. Further logistic regression analyses (Fig. 2b-e) revealed negative relationships between metabolic disorders and HbAA. Conversely, the HbGA/HbAA ratio showed a significantly positive relationship. These results suggested a close association between AA biomarkers and metabolic disorders.

We next performed complex sample logistic regression analysis with increasing degrees of adjustment to explore the associations between HbAA, HbGA, or HbGA/HbAA and NAFLD (Table 2). In a minimally adjusted model with demographic factors taken in, the estimated risk of NAFLD decreased with increasing HbAA quartiles (*P* for trend = 0.001). The ORs (95% CIs) of the increasing quartiles were 0.96 (0.73–1.25), 0.66 (0.52–0.85), and 0.69 (0.52–0.91) compared with the lowest quartile. Consistently, this negative association remained significant after adjustment for risk factors in model 2 [ORs (95% CIs): 0.92 (0.70–1.21), 0.65 (0.50–0.85), and 0.64 (0.48–0.84); *P* for trend < 0.001] and model 3 [ORs (95% CIs): 0.90 (0.68–1.19), 0.65 (0.49–0.85), and 0.61 (0.46–0.81); *P* for trend < 0.001]. In contrast, HbGA/HbAA showed a significantly positive correlation with the risk of NAFLD in model 1 [ORs (95% CIs): 1.69 (1.34–2.13), 2.47 (2.02–3.02), and 2.77 (2.15–3.57); *P* for trend < 0.001]. Similarly, this association remained significant after adjustment for risk factors in model 2 [ORs (95% CIs): 1.60 (1.25–2.04), 2.24 (1.84–2.73), and 2.72 (2.09–3.53); *P* for trend < 0.001] or model 3 [ORs (95% CIs): 1.68 (1.34–2.11), 2.33 (1.85–2.94), and 2.72 (2.06–3.58); *P* for trend < 0.001]. In addition, we did not find a significant association between HbGA and the risk of NAFLD in the adjusted models. In mediation analysis (Table 3), we found that BMI was estimated to explain 59.0% (95%CI: 42.4%–73.8%, *P* < 0.001) of the association between HbAA and NAFLD, while fasting glucose and insulin level could explain 20.5% (95%CI: 13.5%–29.8%, *P* < 0.001) and 68.8% (95%CI: 45.6%–85.2%, *P* < 0.001), respectively. However, no significant intermediating effect was found for blood pressure. Taken together, HbAA is negatively, whereas HbGA/HbAA is positively, associated with the risk of NAFLD. BMI and insulin mediated the relationship between AA and NAFLD.

**Table 2:** Association of AA exposure with the risk of NAFLD in whole group (*n* = 3234)

	HbAA		HbGA		HbGA/HbAA	
	OR (95%CI)	*P*_trend_	OR (95%CI)	*P*_trend_	OR (95%CI)	*P*_trend_
**Model 1**		0.001		0.145		< 0.001
**Q1 (reference)**						
**Q2**	0.96 (0.73–1.25)		1.24 (0.88–1.74)		1.69 (1.34–2.13)	
**Q3**	0.66 (0.52–0.85)		1.27 (0.95–1.70)		2.47 (2.02–3.02)	
**Q4**	0.69 (0.52–0.91)		1.28 (0.98–1.67)		2.77 (2.15–3.57)	
**Model 2**		< 0.001		0.262		< 0.001
**Q1 (reference)**						
**Q2**	0.92 (0.70–1.21)		1.25 (0.89–1.76)		1.60 (1.25–2.04)	
**Q3**	0.65 (0.50–0.85)		1.22 (0.88–1.70)		2.24 (1.84–2.73)	
**Q4**	0.64 (0.48–0.84)		1.24 (0.93–1.64)		2.72 (2.09–3.53)	
**Model 3**		< 0.001		0.998		< 0.001
**Q1 (reference)**						
**Q2**	0.90 (0.68–1.19)		1.07 (0.76–1.49)		1.68 (1.34–2.11)	
**Q3**	0.65 (0.49–0.85)		1.10 (0.79–1.53)		2.33 (1.85–2.94)	
**Q4**	0.61 (0.46–0.81)		1.15 (0.85–1.55)		2.72 (2.06–3.58)	

Quartiles of HbAA: Q1, HbAA ≤ 36.10 pmol/g Hb; Q2, 36.10 < HbAA ≤ 47.60 pmol/g Hb; Q3, 47.60 < HbAA ≤ 66.50 pmol/g Hb; and Q4, HbAA > 66.50 pmol/g Hb

Quartiles of HbGA: Q1, HbGA ≤ 32.30 pmol/g Hb; Q2, 32.30 < HbGA ≤ 44.90 pmol/g Hb; Q3, 44.90 < HbGA ≤ 65.00 pmol/g Hb; and Q4, HbGA > 65.00 pmol/g Hb

Quartiles of HbGA/HbAA: Q1, ≤ 0.732; Q2, 0.732 < HbGA/HbAA ≤ 0.895; Q3, 0.895 < HbGA/HbAA ≤ 1.091; and Q4, HbGA/HbAA > 1.091

Model 1 was adjusted for demographic factors (age, gender, race/ethnicity, education levels, family PIR, marital status, and serum cotinine level)

Model 2 was adjusted for model 1 plus total fat intake and total energy intake

Model 3 was further adjusted for model 2 plus biochemistry factors (serum albumin, LDL-cholesterol, total cholesterol and blood platelet count)

**Table 3:** Results of mediation analysis between HbAA and NAFLD

**Mediator**	**Proportion (%) of effect due to mediation (95% CI)**	***P* value**
**Body fat index**	59.0% (42.4%–73.8%)	< 0.001
**Fasting glucose**	20.5% (13.5%–29.8%)	< 0.001
**Fasting insulin**	68.8% (45.6%–85.2%)	< 0.001
**Blood pressure**^**a**^	2.3% (0.2%–24.8%)	0.226

^a^Blood pressure was denoted as mean arterial pressure

The results of subgroup analyses are shown in Table 4. There was no particular subgroup that showed the strongest negative association between HbAA and NAFLD in socio-economic factors. However, liver function (evaluated by ALT) was detected as a significant modifier for the association (*P* for interaction < 0.001). Stronger negative associations of HbAA with NAFLD were detected among people who had elevated ALT levels [OR_Q4vsQ1_: 0.74 (0.54–1.01) for ALT ≤ 40 U/L and 0.32 (0.11–0.93) for ALT > 40 U/L].

**Table 4:** Odds ratios for associations between HbAA and NAFLD in subgroups

**Subgroups**	HbAA quartile (pmol/g Hb)^a^			*P*_trend_	*P*_interaction_
	Q2 (36.10 < HbAA ≤ 47.60)	Q3 (47.60 < HbAA ≤ 66.50)	Q4 (HbAA > 66.50)		
**Age**					0.130
≤ **50 years**	0.93 (0.56–1.55)	0.80 (0.51–1.26)	0.66 (0.41–1.06)	0.032	
> **50 years**	0.87 (0.66–1.13)	0.51 (0.37–0.70)	0.51 (0.34–0.78)	0.001	
**Gender**					0.717
**Male**	0.95 (0.68–1.31)	0.75 (0.48–1.16)	0.64 (0.36–1.11)	0.102	
**Female**	0.86 (0.60–1.23)	0.55 (0.39–0.75)	0.60 (0.36–1.01)	0.003	
**Race**					0.006
**Non-Hispanic white**	0.82 (0.56–1.19)	0.56 (0.41–0.78)	0.51 (0.37–0.72)	< 0.001	
**Non-Hispanic black**	1.03 (0.40–2.65)	1.06 (0.46–2.41)	0.58 (0.28–1.18)	0.228	
**Mexican American**	0.77 (0.40–1.51)	0.46 (0.26–0.83)	0.69 (0.31–1.56)	0.115	
**Education levels**					0.895
**Under high school**	1.57 (0.76–3.24)	0.63 (0.34–1.16)	0.80 (0.52–1.21)	0.017	
**High school**	0.95 (0.55–1.62)	0.66 (0.39–1.11)	0.63 (0.30–1.34)	0.131	
**Above high school**	0.76 (0.49–1.19)	0.64 (0.42–0.98)	0.53 (0.35–0.78)	0.005	
**Married**					0.887
**No**	1.00 (0.67–1.47)	0.59 (0.36–0.98)	0.65 (0.45–0.92)	0.007	
**Yes**	0.87 (0.63–1.22)	0.67 (0.49–0.90)	0.60 (0.42–0.88)	0.006	
**ALT**					< 0.001
≤ **40 U/L**	0.96 (0.69–1.34)	0.62 (0.45–0.86)	0.74 (0.54–1.01)	0.006	
> **40 U/L**	0.45 (0.16–1.28)	0.36 (0.19–0.66)	0.32 (0.11–0.93)	0.014	

^a^ORs were compared to quartile1 (HbAA ≤ 36.10 pmol/g Hb), adjusted for full model

It has been reported that dietary intake and smoking are generally two important non-occupational sources of AA. To exclude the confounding influence of smoking and to focus on dietary factors, we confined the analysis to the non-smoker group (serum cotinine level ≤ 10 ng/ml) [33]. Similar to the whole group, a negative association between HbAA and NAFLD and a positive association between HbGA/HbAA and NAFLD were found in model 1 and model 2 (Table 5). After adjustment for covariables in model 3, the significant associations remained in the HbAA and HbGA/HbAA groups [ORs (95% CIs): 0.79 (0.63–0.99), 0.72 (0.52–0.99), and 0.57 (0.36–0.88), *P* for trend = 0.014; 1.77 (1.26–2.49), 2.54 (1.71–3.75), and 3.31 (2.18–5.03), *P* for trend < 0.001, respectively]. However, we did not observe a significant association between HbGA and NAFLD in the non-smoker group in model 3. To conclude, AA biomarkers remained related to NAFLD in the non-smoker group.

**Table 5:** Association of AA exposure with risk of NAFLD in non-smokers (*n* = 2620)

	HbAA		HbGA		HbGA/HbAA	
	OR (95%CI)	*P*_trend_	OR (95%CI)	*P*_trend_	OR (95%CI)	*P*_trend_
**Model 1**		0.006		0.111		< 0.001
**Q1 (reference)**						
**Q2**	0.80 (0.64–1.00)		0.93 (0.74–1.15)		1.86 (1.38–2.50)	
**Q3**	0.78 (0.58–1.05)		1.12 (0.93–1.34)		2.63 (1.93–3.59)	
**Q4**	0.57 (0.40–0.82)		1.20 (0.88–1.64)		3.42 (2.40–4.87)	
**Model 2**		0.004		0.205		< 0.001
**Q1 (reference)**						
**Q2**	0.79 (0.62–1.00)		0.93 (0.74–1.18)		1.85 (1.34–2.56)	
**Q3**	0.72 (0.52–0.99)		1.08 (0.87–1.34)		2.61 (1.85–3.68)	
**Q4**	0.54 (0.36–0.81)		1.18 (0.84–1.65)		3.64 (2.44–5.44)	
**Model 3**		0.014		0.325		< 0.001
**Q1 (reference)**						
**Q2**	0.79 (0.63–0.99)		0.79 (0.60–1.04)		1.77 (1.26–2.49)	
**Q3**	0.72 (0.52–0.99)		0.95 (0.76–1.18)		2.54 (1.71–3.75)	
**Q4**	0.57 (0.36–0.88)		1.08 (0.80–1.45)		3.31 (2.18–5.03)	

Quartiles of HbAA: Q1, ≤ 35.00 pmol/g Hb; Q2, 35.00 < HbAA ≤ 44.20 pmol/g Hb; Q3, 44.20 < HbAA ≤ 55.60 pmol/g Hb; and Q4, > 55.60 pmol/g Hb

Quartiles of HbGA: Q1, ≤ 30.30 pmol/g Hb; Q2, 30.30 < HbGA ≤ 41.00 pmol/g Hb; Q3, 41.00 < HbGA ≤ 55.50 pmol/g Hb; and Q4, > 55.50 pmol/g Hb

Quartiles of HbGA/HbAA: Q1, ≤ 0.768; Q2, 0.768 < HbGA/HbAA ≤ 0.926; Q3, 0.926 < HbGA/HbAA ≤ 1.118; and Q4, > 1.118

Model 1 was adjusted for demographic factors (age, gender, race/ethnicity, education levels, family PIR, and marital status)

Model 2 was adjusted for model 1 plus total fat intake and total energy intake

Model 3 was further adjusted for model 2 plus biochemistry factors (serum albumin, LDL-cholesterol, total cholesterol and blood platelet count)

## Discussion

To the best of our knowledge, this is the first study to focus on the association between acrylamide hemoglobin adducts and NAFLD in a representative sample of American adults. The overall prevalence of NAFLD was 30.8%, which is consistent with the prevalence of NAFLD in the U.S. adult population [1]. In this study, we found a negative correlation of serum HbAA levels with NAFLD as well as with metabolic disorders. We also found that the HbAA level remained negatively associated with the risk of NAFLD, yet the HbGA/HbAA level was positively associated with the risk of NAFLD, in the whole group after adjustment for socio-demographic factors, lifestyle factors, and risk factors. Besides, our results showed that HbGA was not significantly associated with the risk of NALFD in the adjusted models.

The neurotoxicity, genotoxicity, and reproductive toxicity of AA have been well demonstrated by previous studies [34–36]. The effects of AA are mediated by the formation of genotoxic metabolites, oxidative stress, affected propagation of neural signals, and interrupted endocrine hormones [37]. In recent years, evidence on its hepatotoxicity has been reported, for the liver is the main site of AA biotransformation. Sun et al. reported that when rats were intraperitoneally injected with 40 mg/kg AA for four weeks, the levels of serum ALT and AST were markedly higher in the treated group than in the normal control group [38]. Additionally, AA (20 mg/kg) increased the levels of liver oxidative stress markers, including protein carbonyl content, nitric oxide, and lipid peroxides, but markedly decreased the activity of liver antioxidants, including superoxide dismutase (SOD) and glutathione peroxidase (GSH-Px). The imbalance between antioxidants and oxidative stress caused the increase of inflammatory markers such as NF-κB, TNF-α, and IL-1β [39, 40]. Furthermore, these stress factors might induce a rapid decline in hepatocyte mitochondrial membrane potential as well as an increase in the activity of caspase-3, the final mediator of apoptosis [41].

However, the results mentioned above were based on AA doses far higher than those the general population is exposed to in daily life. The median HbAA level in our study was 50.6 pmol/g Hb, equivalent to a total uptake of 2 µg/kg body weight/day according to the pharmacokinetics parameters proposed by Calleman [42]. In terms of exposure via food, U.S. Food and Drug Administration (FDA) investigators estimated that the mean and 90^th^ percentile of daily dietary AA exposure of the U.S. population were 0.44 and 0.95 µg/kg body weight [43]. In contrast, it was reported by the Joint Expert Committee on Food Additives (JECFA) that the no observed adverse effect level (NOAEL) of AA for a non-carcinogenic end-point was 0.2 mg/kg body weight/day. According to the above standard, Liu et al. did not discover any toxic effect on rat livers from the metabolic perspective after chronic exposure to AA at NOAEL for 16 weeks [44]. Moreover, in October 2000, the National Toxicology Program (NTP) organized an independent and open peer review and proposed a concept of “low-dose effects,” which referred to the biological changes that occur in the range of human exposures or at doses lower than those typically used in the standard testing paradigm for evaluating reproductive and developmental toxicity [45]. Therefore, whether long-term, low-dose exposure to AA has negative effects on human health needs further investigation.

In recent years, a consensus-driven term “metabolic-associated fatty liver disease” (MAFLD) has been proposed to refine the scope of NAFLD [46]. This updated nomenclature better reflects the underlying metabolic dysfunction that primarily drives this disease [47]. Accordingly, there are some cross-sectional studies utilizing the NHANES database to investigate the relationships between AA and metabolic dysfunction. HbAA was found to be inversely associated with obesity and android fat mass with statistical significance [12, 48]. In addition, Lin et al. [20] reported that increased HbAA was associated with decreased levels of blood insulin and the insulin resistance status in adults. Similarly, we also discovered a negative association between HbAA and the prevalence of metabolic disorders. Since the elements above are common comorbidities of NAFLD, it is reasonable that HbAA was negatively associated with NAFLD in our findings. Indeed, AA has been considered as a potential endocrine-disrupting chemical (EDC) related to various metabolic abnormalities [11]. However, rather than a traditional threshold model or a linear non-threshold model, EDCs may show a U-shaped dose–response curve, commonly called hormesis [49], which means it exhibits a change in the sign of its first derivative [50]. As mentioned above, people’s normal daily exposure to AA is far lower than the NOAEL, and may be located at the decreasing interval of the curves [51]. Grünwald et al. [52] reported that compared with the control group fed flour only, beetles fed flour enriched with 5% charred toast (providing AA) survived significantly longer. This increased stress resistance might result from the activation of arylhydrocarbon receptor (AHR) and nuclear factor erythroid 2-related factor 2 (NRF-2). Nevertheless, the underlying mechanisms need further elucidation.

CYP2E1 plays a crucial role in AA metabolism, and scientists have identified a complex interplay between CYP2E1 and NAFLD [7]. On the one hand, liver CYP2E1 level is inducible, and can be upregulated by high levels of fatty acids, low levels of adiponectin, and the status of insulin resistance [53, 54], which all pathophysiologically characterize NAFLD. Thus, NAFLD patients are likely to have increased CYP2E1 expression and activity [55]. On the other hand, this association can go both ways, that is, higher CYP2E1 activity could also favor liver injury. For example, enhanced enzymatic activity of CYP2E1 promoted reactive oxidative stress (ROS) production, resulting in oxidative stress and hepatic steatosis in mice and LO_2_ cells [56]. In addition, ROS induced mitochondrial dysfunction, a major risk factor in the development of NAFLD, through post-translational protein modifications and mitochondrial DNA damage [57, 58]. O’Shea et al. observed that two pharmacokinetic parameters, mean oral clearance and apparent volume of distribution of chlorzoxazone (mediated primarily by CYP2E1), were about 50% greater in the obese group, consistent with the induction of CYP2E1. Accordingly, such individuals may be at an increased risk of CYP2E1-mediated toxicities and adverse effects [59]. Correspondingly, we found in this study that HbGA/HbAA, which reflects the extent of AA biotransformation, was positively associated with NAFLD in the fully adjusted model. Previous studies reported that the metabolic conversion rate of AA to GA was saturated at low doses because of the saturated activity of CYP2E1 enzyme [60, 61]. This indicated that HbGA/HbAA correlated negatively to HbAA, so the inverse association between HbAA and NAFLD in this study could be a reflection of CYP2E1 activity without AA itself playing a causative role. Ghanayem et al. reported that AA-induced genotoxicity was absent in CYP2E1-null mice [62]. As a result, CYP2E1 is considered to be responsible for AA-related hepatotoxicity.

In addition to CYP2E1, other possible enzymes responsible for AA degradation include microbial amidases, which catalyze the hydrolysis of AA to ammonia and acrylic acid [63]. Amidases also play an important role in cell proliferation. It is noteworthy that some native microbes in the intestines, such as *Escherichia coli* and *Enterococcus faecalis*, may present this potential [64]. There is mounting evidence that the gut and the liver have multiple interactions with each other, and disturbance of the gut–liver axis is linked to NAFLD via complicated mechanisms [65]. Despite a lack of studies that focused on how AA impacts gut microbiota, we believe that it will be a novel research direction.

In subgroup analyses, we revealed a stronger reverse association between HbAA and NAFLD in the elevated ALT (> 40U/L) group. Assay of the serum ALT level has become a primary screening method for liver injury [66]. According to the “multiple hit” hypothesis, NAFLD is a complex disease with multiple insults acting together [67]. Therefore, we may keep an eye on the AA level of people with liver dysfunction so as to avoid an extra hit by AA.

Although we used a large-scale, quality-controlled database to draw conclusions, there exist some limitations in our study. First, owing to the cross-sectional nature of this study, it was not possible to infer directly which factor came first. In this study, we discovered a negative association between HbAA and NAFLD. However, it seems more reasonable that this association is a reflection of the CYP2E1 activity, that is, HbGA/HbAA, rather than an indication that AA is responsible for the development of NAFLD. Regretfully, we did not acquire the information about the expression or polymorphisms of CYP2E1 in the current study population. Furthermore, French fries and processed cereal-grain-based foods were discovered to be the greatest contributors to dietary AA intake, while fresh vegetables and fruits were just the opposite [43]. As restricting high-carbohydrate foods and consuming more vegetables and fruits are recommended for NAFLD patients [68], another possible explanation for the negative association is that people with NAFLD might have followed dietary advice to restrict the consumption of foods containing AA. Second, NAFLD was determined by socio-demographical data and blood biomarker values, in which misclassification bias might be inevitable. In addition, to avoid overcorrection, we had to exclude overweight and diabetes classifications from the regression models, although it would be of interest to assess whether these conditions mediated the association between AA and NAFLD. Third, AA Hb biomarkers can only reflect the exposure level within 120 days, which is not long enough to estimate the cumulative effect of AA exposure across several years [8]. Therefore, further long-term cohort studies should be employed to elucidate these relationships.

## Conclusion

In conclusion, we reported that HbAA was negatively and HbGA/HbAA was positively associated with NAFLD in the U.S. population, independently of traditional NAFLD risk factors. Further studies are needed to clarify the relationships between them.

## Data Availability

The datasets analyzed during the current study are available in the NHANES repository, https://wwwn.cdc.gov/nchs/nhanes/default.aspx.

## Abbreviations

AA: Acrylamide
ALT: Alanine aminotransferase
BMDL10: Benchmark dose lower confidence limit
BMI: Body mass index
CI: Confidence interval
EDC: Endocrine-disrupting chemical
EFSA: European food safety authority
FDA: Food and Drug Administration
GA: Glycidamide
GGT: Gamma glutamyl transferase
GSH: Glutathione
Hb: Hemoglobin
HbA1c: Glycated hemoglobin
HbAA: Hemoglobin adducts of acrylamide
HbGA: Hemoglobin adducts of glycidamide
HBV: Hepatitis B virus
HCV: Hepatitis C virus
HDL-C: High density lipoprotein cholesterol
HPLC/MS/MS: High-performance liquid chromatography tandem mass spectrometry
JECFA: Joint Expert Committee on Food Additives
LDL-C: Low density lipoprotein cholesterol
MOE: Margin of exposure
NAFLD: Non-alcoholic fatty liver disease
NHANES: National Health and Nutrition Examination Survey
NOAEL: No observed adverse effect level
NTP: National Toxicology Program
OR: Odds ratio
PIR: Income-to-poverty ratio
ROS: Reactive oxidative stress
SOD: Superoxide dismutase
T2DM: Type 2 diabetes mellitus
TG: Triglyceride
US FLI: U.S. fatty liver index
